# Seroepidemiology of bluetongue in South Bengal

**DOI:** 10.14202/vetworld.2016.1-5

**Published:** 2016-01-02

**Authors:** Arkendu Halder, Siddhartha N. Joardar, Devi Prasad Isore, Indranil Samanta, Panchanan Parui, Dhriti Banerjee, Chandan Lodh

**Affiliations:** 1 Department of Veterinary Microbiology, West Bengal University of Animal and Fishery Sciences, Kolkata - 700037, West Bengal, India; 2 Diptera Section, Zoological Survey of India, Kolkata - 700059, West Bengal, India; 3 Department of Veterinary Medicine, Ethics and Jurisprudence, West Bengal University of Animal and Fishery Sciences, Kolkata - 700037, West Bengal, India

**Keywords:** antibodies, bluetongue, *Culicoides* sp, enzyme linked immunosorbent assay, South Bengal

## Abstract

**Aim:**

With the aim of revealing the epidemiological intricacies of bluetongue (BT) in the southern part of West Bengal state, the present study was undertaken to assess seroprevalence of BT along with identification of the vector of the disease, i.e., *Culicoides* midges available in the region in their breeding season with conducive environmental factors, if any.

**Materials and Methods:**

A total of 1509 (sheep-504, goat-1005) samples were collected from three different agroclimatic zones of South Bengal *viz*. new alluvial, red laterite and coastal saline. To detect anti-BT antibodies in the collected serum samples, indirect-enzyme-linked immunosorbent assay (i-ELISA) was performed. *Culicoides* midges were collected from those agro-climatic zones of South Bengal for species identification. The meteorological parameters, *viz*. temperature (maximum and minimum), rainfall and relative humidity of three agro-climatic zones of South Bengal were analyzed for the months of July to December during 2010-2013.

**Results:**

The overall seropositivity was 33.13% and 30.24% in sheep and goat, respectively as assessed by i-ELISA. In South Bengal, the predominant species of *Culicoides* found were *Culicoides schultzei, Culicoides palpifer* and *Culicoides definitus*.

**Conclusion:**

Since virus transmitting species of *Culicoides* midges could be detected in South Bengal, besides high seropositivity in ruminants, the possibility of circulating BT virus in South Bengal is quite imminent.

## Introduction

Bluetongue (BT) is an acute, infectious, arthropod-borne viral disease of a wide range of domestic and wild ruminants. The major hosts of BT are sheep and some wild ruminants. Cattle, goat and some other wild ruminants show the disease subclinically [1]. BT is the member of genus Orbivirus of the family Reoviridae [2]. The epizootics of the disease depend on the complex interaction of host, vector (*Culicodes* midges) and virus [3].

In the eastern part of India, the incidence of BT is not detected so far unlike southern and western parts [4,5]. However, anti-BT antibodies could be detected in sheep, goat, and cattle population of one of the eastern Indian state, West Bengal [6]. Detection of anti-BT antibodies in the host is the indirect evidence of the presence of virus in that specific geographical region [7]. West Bengal state is divided into six different agro-climatic zones *viz*. hilly, tarai, old alluvial, new alluvial, red laterite and coastal saline.

**Figure-1:**
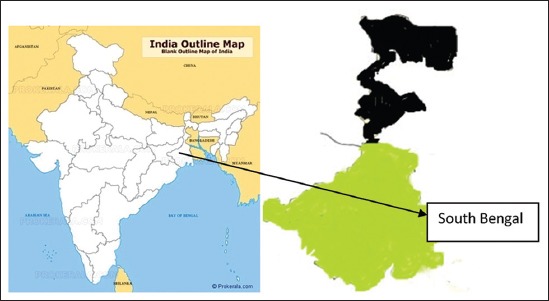
Study area- South Bengal (southern part of West Bengal state) depicted as green color.

In this present study, the southern part of the river Ganga and that of the state (known as South Bengal, Figure-1) which comprises three agroclimatic zones *viz*. New Alluvial, Red Laterite and Coastal Saline were considered for serum sample collection. To reveal the epidemiological intricacies of BT in the region, the present study was undertaken to assess seroprevalence of BT along with identification of the vector of the disease, i.e. *Culicoides* midges available in the region in their breeding season with conducive environmental factors, if any, in a holistic manner that has not been done in any earlier study.

## Materials and Methods

### Ethical approval

As per CPCSEA guidelines, study involving clinical samples does not require approval of Institute Animal Ethics Committee. However, samples were collected as per standard sample collection methods without any harm or stress to the animals.

### Serum samples

Blood samples without anti-coagulant were collected from suspected animals, *viz*. sheep and goat of the targeted agro-climatic zones during the month of July to December, from 2010 to 2013. A total of 1509 (sheep-504, goat-1005) samples were collected from three different agro-climatic zones *viz*. new alluvial, red laterite and coastal saline of South Bengal (Table-1). Sera were separated and stored at−20°C. Serum samples were screened by indirect-enzyme-linked immunosorbent assay (i-ELISA) for detecting anti-BT antibodies.

**Table-1:** Serum samples collected from different agro-climatic zones of West Bengal during July, 2010-December, 2013.

Agroclimatic zone	Animal species	Number of samples
New alluvial	Sheep	122
	Goat	332
Red laterite	Sheep	250
	Goat	120
Coastal saline	Sheep	132
	Goat	120

### i-ELISA

To detect anti-BT antibodies in the collected serum samples, i-ELISA was done using i-ELISA kit, procured from the eastern regional collaborating center of All India Network Programme on BT (AINP-BT, Kolkata center) of Indian Council of Agricultural Research. The test was performed as per Joardar *et al*. [5]. Reading was taken in an ELISA plate reader (ECIL, India) at 492 nm. The average optical density value of negative control was calculated and compared with the test samples.

### Culicoides vector

*Culicoides* midges were collected from three agro-climatic zones of West Bengal (red laterite, coastal saline and new alluvial) for species identification. The midges were collected during the months covering post-monsoon to the winter season in the region as the number and activity of *Culicoides* midges remains rampant at that time. The midges were trapped in the early evening, sorted out and stored in ethanol until used for identification.

### Identification of Culicoides midges

Midges of *Culicoides* species were identified based on morphological characteristics [8,9].

### Metereological parameters

The meteorological parameters, *viz*. temperature (maximum and minimum), rainfall and relative humidity of three agro-climatic zones of South Bengal were collected from Regional Meteorological Center, Alipore, Kolkata for the months of July to December during 2010-2013 and analyzed to assess whether conducive environment exists in South Bengal for the propagation of *Culicoides* midges or not.

## Results

### Assessment of sheep serum samples by i-ELISA

When the sheep sera were assessed by i-ELISA, it was found that out of 504 samples, 167 samples were positive in i-ELISA. The overall seropositivity was 33.13%. The detail of the result is given in Table-2.

**Table-2:** Assessment of sheep serum samples by i-ELISA to detect anti-bluetongue antibodies.

Agro-climatic zone	Number of sample collected	Number of positive samples	Number of negative samples	% positivity
New alluvial	122	48	74	39.34
Red laterite	250	79	171	31.60
Coastal saline	132	40	92	30.30
Total	504	167	337	33.13

ELISA: Enzyme linked immunosorbent assay

### Assessment of goat serum samples by i-ELISA

When i-ELISA was done with the serum samples collected from goat, it was found that out of 1005 samples, 304 samples (30.24%) were positive. The detail result of i-ELISA is given in Table-3.

**Table-3:** Assessment of goat serum samples by i-ELISA to detect anti-bluetongue antibodies.

Agro-climatic zone	Number of samples collected	Number of positive samples	% positivity
New alluvial	332	137	41.26
Red laterite	553	120	21.69
Coastal saline	120	47	37.16
Total	1005	304	30.24

ELISA: Enzyme linked immunosorbent assay

### Identification of Culicoides midges

*Culicoides* midges those were collected from different parts of South Bengal were identified up to species level based on morphological characteristics. The midges were medium sized flies with moderately hairy wings with numerous distinct pale spots including a pale spot over r-m crossvein almost on the center of the vein. Radial cells were absent; anterior border of wing with two dark spots, one at the tip of radial vein forming the stigma, other at about the middle of cell R5. Aedaegus was prominent with saddle-shaped stem. Paramere was broad at the base, curved and tapered toward the tip with apical hairs. The identification results are given in Table-4.

**Table-4:** Identification of *Culicoides* species from different areas of South Bengal.

Area of collection	Agro-climatic zone	Month of collection	Species identified
Khatra (District-Bankura)	Red laterite	April	*C. schultzei*
Belgachia (District-Kolkata)	New alluvial	November	*C. schultzei, C. palpifer, C. definitus*
Agarpara (District-North 24 pgs)	New alluvial	December	*C. schultzei*
Kamarhati (District-North 24 pgs)	New alluvial	January	*C. schultzei*
Sandeshkhali (District-North24 pgs)	Coastal saline	January	*C. schultzei*
Patelnagar (Disttict-Birbhum)	Red latterite	August	*C. schultzei*
Hariharpara (District-Murshidabad)	New alluvial	November	*C. schultzei*
Malda town (District-Malda)	New alluvial		*C. schultzei*

C. schultzei: Culicoides schultzei, C. palpifer: Culicoides palpifer, C. definitus: Culicoides definitus

### Metereological parameters

Temperature (maximum and minimum), rainfall and relative humidity of three agro-climatic zones were collected and analyzed to know the environmental condition of South Bengal at the collection time (Table-5). Maximum temperature varied from 31 to 38°C and minimum temperature varied in between 12 and 24°C. Relative humidity ranged from 72% to 86% and average rainfall varied within therange of 4-307 mm.

**Table-5:** Meteorological parameters of the study areas (agro-climatic zones) during collection period (July, 2010- December, 2013).

Agro-climatic zone	Temperature (°C)	Relative humidity (%)	Rainfall (mm)
New alluvial	Maximum-32 Minimum -12	72-85	42-50
Red laterite	Maximum -38 Minimum -24	73-86	55-276
Coastal saline	Maximum -31 Minimum -12	80-86	83-07

## Discussion

With the aim of exploring the BT epidemiology in South Bengal, attempts were made to correlate the presence of anti-BT antibodies in small ruminants (sheep and goat) of different agro-climatic zones along with the prevalence of *Culicoides* midges in those areas.

In the present study conducted during 2010-2013, when sheep sera were assessed by i-ELISA, it was found that 33.13% serum samples possessed anti-BT antibodies. In the case of goat, the percentage was 30.24. In one earlier study, similar seropositivity (34.47%) was reported in sheep of South Bengal [10]. However, lower % positivity in goat (24.03%) and cattle (16.21%) were observed in the same study. Considerable low seropositivity (2.69% and 2.13%) was reported in ruminants of Central Iran and South-east Iran, respectively [11,12].

New alluvial, red laterite and coastal saline zones of West Bengal were taken into consideration and the seroprevalence detected in sheep were 39.34%, 31.60% and 30.30%, respectively. The seroprevalence ranged from 30% to 40% in different zones that indicates the prevalence of the virus in all those agro-climatic zones of West Bengal. Earlier, comparable seroprevalence (overall 42.31%) was reported in Andhra Pradesh [13]. However, the present report does not corroborate with the results of south Indian states. An overall 71.43% seroprevalencewas observed in three states of South India, with 65.19% in Andhra Pradesh, 79.5% in Karnataka and 80.95% prevalence in Tamil Nadu using competitive ELISA (cELISA) [14]. However, low seroprevalence (9.3%) was reported in ruminants of Kerala [15].

In this study, 33.13% seroprevalence was observed in sheep. Though, a much higher (87%) report of seroprevalance from Tamil Nadu [16] and a much lower (23.5%) report from Haryana, Himachal Pradesh and Punjab [17] were also observed. In Maharashtra state, 40.36% seropositivity was reported in 2006 [18]. In sheep, 36.11% and 30.3% seropositivitywere reported from Gujarat in 2004 [19] and 2005 [20], respectively. Shlash Khalid *et al*. [21] found 43.97% seropositivity in sheep of Iraq using cELISA.

The overall seropositivity in goat was nearly equal to sheep which is in opposite with earlier findings [22]. Goats, though refractorymay be an unapparent host to the virus and an important link in the epidemiology of the disease [23]. In India, most sheep are grazed and housed with goats and the implications of these mixed flocks on the epidemiology and control of the disease are profound and need to be investigated in more detail [24].

In another recent study, when total 364 animal sera (sheep-120, goat-112, cattle-132) were screened by i-ELISA, 32 of sheep (26.66%), 35 of goat (31.25%), and 69 (52.27%) of cattle samples were found positive, though no outbreak or incidence of BT in animals of Orissa being reported so far [25]. The percent positivity of sheep and goat serum collected from Orissa was similar with the values of serum samples collected in the present study in South Bengal. Surprisingly, no outbreak occurred in this state though a considerable number of small ruminants show high titer of anti-BT antibodies.

*Culicoides* midges were collected from different agro-climatic zones of South Bengal and identification upto species level was carried out. *Culicoides* species identified from three agro-climatic zones (new alluvial, red laterite and coastal saline) were *Culicoides schultzei*, *Culicoides palpifer*, and *Culicoides definitus*. However, in an earlier study *Culicoides actoni, Culicoides clavipalpis*, *Culicoides oxystoma*, *Culicoides anopheles*, *C. palpifer* and *Culicoides alatus* were identified belonged to certain agro-climatic zones of West Bengal [26]. In Indonesia during 1993, serotype 21 of BT virus was isolated from *C. palpifer* [27]. *Culicoides brevitarsis* Keiffer and *C. imicola* Keiffer are proven vectors of BT virus (BTV), occurring widely in India [28]. Recently, BTV (serotype-16) could be isolated from *C. schultzei* trapped from Kamarhati area (24 pgs District of South Bengal) [29]. The *C. schultzei* complex contains several species *viz*. *C*. *oxystoma*, *C*. *schultzei*, *Culicoides subschultzei*, *Culicoides kingi*, *Culicoides rhizophorensis*, *Culicoides enderleini*, *Culicoides nevilli* and *Culicoides neoschultzei* [30,31] having very less morphological differences. Of these midges, *C*. *oxystoma* and *C*. *schultzei* were proven as potential vectors for BTV and epizootic hemorrhagic disease virus responsible for transmitting the virus among different groups of animals [32].

In new alluvial region, the average maximum temperature was more than 25°C, except the month of December. In the case of red laterite area, the average maximum temperature was more than 28°C and in thecoastal saline region it was more than 30°C except the month of December when maximum temperature was 25°C. It is an established fact that the spread of BTVis closely related with air temperature; atemperature between 19 and 32°C being most conducive for transmission. Temperature below 9°C inhibits the virus replication, depending on the strain of virus concerned [33]. It was also noticed that at a higher temperature, a greater proportion of *Culicoides* midges would be competent to transmit BTV and some refractory species of *Culicoides* (*C. nubeculosis*) become competent in favorable atmospheric condition [34]. From the data shown in Table-5, it is clear that the atmospheric temperature in those three agro-climatic zones favors the replication of the virus in the vector. The relative humidity of those agro-climatic zones varied from 73% to 86%. The relative humidity required for propagation of midges is more than 80%. Hence, it can be inferred that the environmental parameters are quite conducive for the propagation of the vectors in South Bengal.

## Conclusion

As virus transmitting species of *Culicoides* midges could be detected in South Bengal, besides high seropositivity in ruminants, the possibility of circulating BTV in South Bengal cannot be ruled out. As such to avoid the menace of BT outbreaks, more surveillance of the disease incidence should be encouraged and general preparedness to counter outbreaks is advocated.

## Authors’ Contributions

Collection of serum samples, screening of samples by ELISA and collection of *Culicoides* midges were done by AH. The midges were identified by PP and DB. The entire work was done under the supervision of SNJ. Data were analyzed by SNJ and CL. The manuscript was prepared by AH and correction/modifications were done by DPI and IS. All authors read and approved the final manuscript.
